# Predictors of Changes in Cognitive Function in Older Korean Adults: The 2006–2018 Korean Longitudinal Study of Aging

**DOI:** 10.3390/ijerph18126345

**Published:** 2021-06-11

**Authors:** Minjeong Kang, Inhwan Lee, Haeryun Hong, Jeonghyeon Kim, Hyunsik Kang

**Affiliations:** College of Sport Science, Sungkyunkwan University, Suwon 16419, Korea; kangmin125@skku.edu (M.K.); ansh00@naver.com (I.L.); hhr8028@skku.edu (H.H.); zzagkim115@naver.com (J.K.)

**Keywords:** handgrip strength, cognition, geriatrics, health behaviors

## Abstract

Cognitive decline with normal aging varies widely among individuals. This study aimed to investigate predictors of longitudinal changes in cognitive function in community-dwelling Korean adults aged 65 years and older. Data from 727 older adults who participated in the Korean Longitudinal Study of Aging (KLoSA) survey from 2006 (baseline) until 2018 (seventh wave) were used. Cognitive performance was assessed with the Korean Mini-Mental State Examination. The participants were retrospectively classified into normal cognition, mild cognitive impairment, and moderate/severe cognitive impairment. Education, income, religion, living area, alcohol intake, smoking, physical activity, handgrip strength, functional dependency, depression, comorbidity, medications, fall experience, and unintentional weight loss were included as covariates. A linear mixed regression analysis showed that a steeper decline in cognitive function over time was significantly associated with parameters of poor socio-economic status, health conditions, and unhealthy behaviors. Individuals with mild cognitive impairment or moderate/severe cognitive impairment were likely to have steeper cognitive declines compared with individuals with normal cognition. The current findings of the study showed that age-related cognitive decline was multifactorial in older Korean adults.

## 1. Introduction

Population aging is an inevitable and global trend due to lower fertility and reduced mortality, especially in developed and developing countries [1]. Since its initial status as an aged society in 2017, South Korea in 2019 comprised a 15.5% population aged 65 years and older and is expected to be a super-aged society by 2060, when 41% of the population is expected to be geriatric [2]. From a Korean public health perspective, such a rapidly growing proportion of older adults implies a substantial burden and cost to individuals and society due to the various health conditions associated with aging [3,4].

Age-related cognitive decline is a priority public health issue because it can be indicative of the preclinical stage of dementia [5,6]. A systematic review and meta-analysis of 11 previous studies involving Korean adults aged 65 years and older showed that the prevalence of dementia was 9.2%, higher than those observed in Western and other Asian populations [7]. By analyzing data extracted from previous national statistics and nationwide studies conducted in South Korea, Park et al. [8] reported that the dementia-related burden of disease was estimated to be 528 per 100,000 people, which was highest in the elderly Korean population and is expected to increase sharply with population aging. Therefore, understanding the etiology of cognitive decline with normal aging is imperative for therapeutic strategies to slow age-related cognitive impairment and reduce the odds of its pathologic progress toward dementia [9].

There is growing evidence that age-related cognitive decline varies widely among individuals, from highly sustained performance to steeply accelerating decline [10], and is influenced by individual differences in risk factors [11]. With respect to etiology, age and dementia are significantly associated with steeper cognitive decline [12]. Behavioral risk factors including heavy alcohol consumption, smoking, physical inactivity, and poor physical fitness, and functional limitations are also associated with steeper cognitive decline with normal aging [13,14]. Low socio-economic status such as poor education and low income and demographic characteristics such as being female or having no spouse are additional predictors of age-related cognitive decline [13,14]. Likewise, previous studies have reported a number of risk factors associated with age-related cognitive decline in older Korean adults, and they include sociodemographic parameters, unhealthy behaviors, and poor health status [15,16,17,18,19,20].

Compared to cross-sectional studies looking at a specific point in time, long-term cohort studies that assess risk factors and characterize changes in cognitive function over time are better for providing insights into the etiology of age-related cognitive decline and identifying its risk factors [21]. This study aimed to explore predictors of longitudinal changes in cognitive function in community-dwelling older Korean adults by analyzing a nationally representative survey data set.

## 2. Materials and Methods

### 2.1. Data Source and Study Participants

The Korean Longitudinal Study of Aging (KLoSA) was a biannually conducted, nationwide survey through computer-assisted personal interviewing between 2006 and 2018 (2006, 2008, 2010, 2012, 2014, 2016, and 2018) in South Korea. The 2006 KLoSA survey was a prospective cohort study that included those who were cognitively normal at baseline. For simplicity purposes, this study analyzed data from 727 participants (54.6% male) aged 65 years and older who completed all the 7 successive waves of the 2006–2018 KLoSA. Sample size was calculated by using statistical power analysis software (effect size = 0.15, statistical power = 0.80, *p* = 0.05; G*power 3.0.10. program, Kiel university, Schleswig-Holstein, Germany). A detailed description of the KLoSA is provided elsewhere [22]. The survey protocol was reviewed and approved by the Institutional Review Board of Statistics Korea (approval number: 336052). All participants provided written informed consent. Access to the KLoSA data is available via the National Public Database (https://survey.keis.or.kr/eng/klosa/klosa01.jsp accessed on 25 January 2021).

### 2.2. Study Variables

#### 2.2.1. Cognitive Function

Cognitive function was measured using the Korean mini-mental state examination (K-MMSE). Briefly, the K-MMSE consists of the following six domains: time and place orientation, registration, attention and calculation, recall, language, and visual construction. The maximum total K-MMSE score is 30 points. Based on individual K-MMSE scores assessed in the seventh wave of the KLoSA survey, the participants were retrospectively classified into three categories: normal cognition (score ≥ 24), mild cognitive impairment (score 20–23), and moderate/severe cognitive impairment (score ≤ 19). Classification of cognitive declines based on K-MMSE scores was previously tested and validated in a sample of patients with Alzheimer’s disease, vascular dementia, and depression [23]. However, chance of making type 2 error (or false negative) was increased at the early stage of cognitive dysfunction, which was another reason why we limited our data analysis to the older adults only.

#### 2.2.2. Covariates

The covariates used in this study included demographics, SES, health behaviors, functional limitations, and health conditions. Sociodemographic factors were age (continuous scale), sex (categorical: male or female), education (categorical: elementary school or less, middle or high school, university or higher), area of residence (categorical: urban or rural), income (quantitative), married (categorical: yes or no), and religion (categorical: yes or no).

Health behavioral factors were smoking (categorical: current/former or never) and heavy alcohol consumption (categorical: yes or no). Heavy alcohol consumption was assessed with the response to the following question: “Do you have 5 or more alcohol drinks per week?” [24]. In addition, the participants were asked to report the frequency and duration of weekly physical activity, and they were categorized as physically inactive (<150 min per week) or physically active (≥150 min per week) [25]. Handgrip strength (kg) was assessed with a handgrip strength dynamometer (TANITA No. 6103, Tokyo, Japan).

Parameters of functional limitations included the Korean Activities of Daily Living (K-ADL) score (continuous scale) and Instrumental Activities of Daily Living (IADL). Higher scores in the K-ADL and IADL scales indicate greater dependency. Parameters of health conditions included depressive symptoms (continuous scale), fall experience (categorical: yes or no), number of physician-diagnosed medical conditions (continuous scale), number of medications (continuous scale), and experience of unintentional weight loss (UWL) of 5 kg or more in the past year (categorical: yes or no) [26]. Depression was measured using the 10-item short-form of the Center for Epidemiological Studies Depression (CESD-10) scale modified Korean version [27].

### 2.3. Statistics

Prior to statistical analyses, normality of data distribution was confirmed with QQ plotting, and absence of multi-collinearity among the predictors was assessed by variance of inflation factors (VIFs). Descriptive statistics, such as frequency (%) and average, standard deviation (SD), and 95% confidence interval of socio-demographics, health behaviors, and health conditions among the three categories of longitudinal cognitive functioning were performed in the first step of the analysis. Then, bivariate correlations between cognitive function and all the measured covariates were calculated. Finally, data were analyzed by using a linear mixed model (LMM) with maximum likelihood (ML) estimation. The aim of the LMM was to estimate the predictors for changes in cognitive function over time on the basis of the data we collected. The model was built by inserting the fixed factors (i.e., all the covariates and three categories of cognitive declines), a random intercept relating to measurement time in the survey, and a random slope on measurement time considered. In addition, an interaction between cognitive function and time was included as an additional explanatory variable in order to test whether categories of cognitive functioning were associated with linear change in cognitive function [28,29]. All statistical analyses were conducted using PASW SPSS WIN 27.0 (SPSS Inc., Chicago, IL, USA), and *p* ≤ 0.05 was considered statistically significant.

## 3. Results

Among the 727 participants who completed the 7 successive waves of the 2006–2018 KLoSA, 55% maintained normal cognition, 20% had mild cognitive impairment, and 25% had moderate/severe cognitive impairment. Table 1 presents the descriptive statistics of the measured parameters according to category of cognitive impairment. At baseline, individuals with mild or moderate/severe cognitive impairment were likely to be older (*p* < 0.001), male (*p* < 0.001), and residents of a rural area (*p* = 0.048) and were likely to have no spouse (*p* = 0.006), lower educational attainment (*p* < 0.001), and lower household income (*p* < 0.001) compared to individuals with normal cognition. In addition, individuals with mild or moderate/severe cognitive impairment had higher rates of heavy drinking (*p* = 0.046), physical inactivity (*p* = 0.005), and UWL (*p* = 0.001), higher numbers of comorbidities (*p* = 0.004) and medications (*p* = 0.011), higher CES-D score (*p* < 0.001), and higher K-ADL score (*p* = 0.015), in conjunction with lower handgrip strength (*p* < 0.001) compared to individuals with normal cognition.

Table 2 presents correlation coefficients between cognitive function and all the measured covariates. In general, cognitive function was significantly related to age (r = −0.193, *p* < 0.001), sex (r = −0.136, *p* < 0.001), existence of spouse (r = −0.085, *p* < 0.001), residence area (r = −0.107, *p* < 0.001), education (r = 0.234, *p* < 0.001), income (r = 0.152, *p* < 0.001), smoking (r = 0.071, *p* < 0.001), heavy drinking (r = 0.063, *p* < 0.001), physical activity (r = 0.069, *p* < 0.001), handgrip strength (r = 0.185, *p* < 0.001), CES-D score (r = −0.114, *p* < 0.001), experience of falls (r = −0.029, *p* = 0.006), number of comorbidities (r = −0.091, *p* < 0.001), number of medications (r = −0.046, *p* < 0.001), and UWL (r = 0.033, *p* = 0.002) but not related to BMI, having a religion, and K-ADL score.

Table 3 presents parameter estimates of longitudinal changes in cognitive function. In the unadjusted model, changes in cognitive function were significantly associated with age (β = −0.045, *p* = 0.003), (urban residence (β = 0.398, *p* = 0.001), elementary education or less (β = −1.159, *p* < 0.001), middle/high school education (β = −0.642, *p* = 0.026), household income (β = 0.001, *p* = 0.017), fall experience (β = −0.682, *p* = 0.008), heavy drinking (β = −0.431, *p* = 0.014), UWL (β = −0.592, *p* = 0.001), K-ADL score (β = −0.183, *p* = 0.042), and handgrip strength (β = 0.034, *p* = 0.004), in conjunction with moderate/severe cognitive impairment (β = −4.524, *p* < 0.001) and mild cognitive impairment (β = −0.245, *p* < 0.001). In addition, there was a decline of 0.494 points in cognitive function every 2 years (*p* < 0.001).

In the adjusted model including an interaction term between cognition category and time, elementary education or less (β = −1.126, *p* < 0.001), middle/high school education (β = −0.605, *p* = 0.037), household income (β = 0.001, *p* = 0.016), UWL (β = −0.735, *p* = 0.004), depression (β = −0.054, *p* = 0.017), and handgrip strength (β = 0.039, *p* = 0.018) remained significant predictors of changes in longitudinal cognitive function. In particular, the interactions between moderate/severe cognitive impairment and time (β = −1.740, *p* < 0.001) and between mild cognitive impairment and time (β = −0.775, *p* < 0.001) were statistically significant, implying that distinct changes in cognitive function can present according to severity of cognitive impairments (from normal cognition to moderate/severe cognitive impairment).

**Figure 1:**
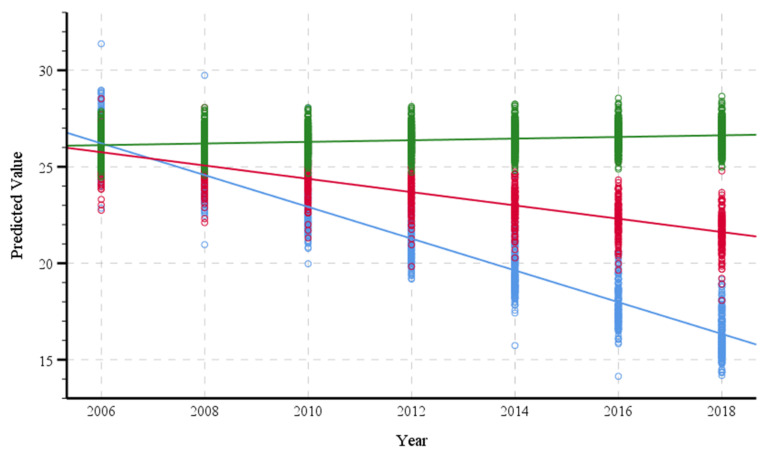
Predicted means of cognitive function in normal cognition (blue), mild cognitive impairment (red), and moderate/severe cognitive impairment (green).

Figure 1 illustrates predicted means of cognitive function over the 12-year follow up by category of cognitive impairment. Individuals with moderate/severe cognitive impairment had a significant decline in cognitive function over time (β = −1.646, *p* < 0.001), as did individuals with mild cognitive impairment (β = −0.690, *p* < 0.001). The slopes for the moderate/severe and mild cognitive impairments were significantly different compared to that of normal cognition (*p* < 0.05 after Bonferroni correction).

## 4. Discussion

In this prospective cohort study, we examined predictors of longitudinal cognitive decline in a sample of community-dwelling Korean older adults and found that steeper cognitive decline was associated with lower educational background, lower household income, UWL, depression, and lower handgrip strength. In particular, individuals with mild cognitive impairment or moderate/severe cognitive impairment were likely to experience a steeper decline in cognitive function over time. Novel to our investigation is the findings that age-related cognitive decline is associated with lower SES, unhealthy behaviors, lower handgrip strength, lower functional capacity, and health conditions in conjunction with predisposition to cognitive impairment in Korean older adults. The current findings of the study suggest that a multimodal intervention targeting lower SES, behavioral risk factors, and health conditions may be a better strategy against cognitive decline with normal aging in Korean older adults.

Consistent with the current findings, previous studies showed that lower educational attainment, lower household income, and living in rural areas were associated with greater cognitive decline with normal aging in older Asian adults [30,31]. In addition, previous studies also reported that gender, lower income, lower functional capacity, and social inactivity were potential modulators and/or predictors for cognitive decline with normal aging in older Korean adults [32,33,34]. In this aspect, findings from the Korean urban rural elderly cohort study, in which 3517 participants aged 65 years and older completed baseline assessments, show that Korean older adults residing in a rural area had lower educational attainment, lower household income, and higher rates of heavy drinking, smoking, and being underweight compared with their counterparts residing in an urban area [35]. By contrast, higher educational attainment, higher household income, and living in advantaged environments were protective of age-related cognitive decline in older adults [36,37].

In addition to poor SES and unhealthy behaviors, existing health conditions such as lower functional capacity [38], UWL [39], and depression [40] contribute to age-related cognitive decline and onset of cognitive impairment [6]. In contrast, there is emerging evidence that healthy lifestyles including physical activity [41] and fitness [42] as well as good nutrition [43] protect against cognitive decline during normal aging. Likewise, the current findings also showed that age-related cognitive decline was positively associated with higher K-ADL score, UWL, and depression but inversely associated with handgrip strength.

In accordance with the current findings, Kang et al. [44] found that cognitive function was negatively associated with age and depression but positively associated with handgrip strength and walking speed in a sample of 121 Korean outpatients aged 65 years and older who registered in a local hospital. Kim et al. [33] showed that handgrip strength at baseline was an independent predictor of age-related cognitive decline in a cohort of 2374 older Korean adults from the 2006–2014 KLoSA. In a nationwide cohort study involving 167,876 newly diagnosed patients with diabetes, Nam et al. [45] found that weight loss >10% from the time of diagnosis of diabetes until 2 years later was associated with an increased risk of Alzheimer’s disease. Together, findings from the current and previous studies suggest that emphasis should be on reducing health disparities associated with SES and behavioral risk factors, enhancing protective factors, and managing health conditions to promote cognitive health in older populations [46].

The current findings of the study also suggest that age-related cognitive decline may be associated with the presence of cognitive impairment in older Korean adults. In agreement with the current findings, Lee et al. [6] found that subjective cognitive decline assessed with the Korean dementia pre-screening questionnaire was significantly associated with increased risk of subsequent dementia in a nationwide population-based cohort study involving 579,710 adults aged 66 years and older. In a multicenter prospective cohort study involving 6105 participants with previous stroke or transient ischemic attack, Rist et al. [47] showed that low baseline cognitive function was significantly associated with increased risk of dementia during an average follow-up period of 3.8 years.

In the present study, however, the directionality of the relationship between longitudinal changes in cognitive function and cognitive impairments cannot be determined. That is, the association between age-related cognitive decline and cognitive impairments might be directional; age-related cognitive decline is inevitable and can contribute to cognitive impairments in many cases, or vice versa. Although the exact mechanisms linking cognitive impairments to age-related cognitive decline remain to be investigated, the findings of the current study suggest that cognitive decline with normal aging can be minimized by prevention, early detection, and treatment of cognitive impairment.

Some explanations for predictors of age-related decline in cognitive function can be offered. First, lower SES in conjunction with living in rural areas may imply geographic isolation, less chance of employment, higher rates of behavioral risk factors and health conditions, and limited access to healthcare services, collectively contributing to cognitive decline and impairments [48,49]. Second, lower handgrip strength may represent an early sign of age-related cognitive decline [50] or overall health status including cognitive dysfunction and depression [51] in geriatric populations. Third, late-life depression itself or by interacting with frailty phenotypes such as UWL, physical inactivity, and lower handgrip strength may an impact on geriatric mental conditions including mild cognitive impairment and dementia [52,53]. Fourth, existing health conditions may accelerate cognitive decline with normal aging and thereby increase the risk for cognitive impairments and dementia [54].

This study has some limitations. First, although K-MMSE score-based classification of cognition categories was previously tested and validated in a sample of Korean patients with Alzheimer’s disease, vascular dementia, and depression [23], the instrument is a subjective screening test and can be erroneous and vulnerable to ceiling effect for normal individuals [55]. Consequently, other options in conjunction with K-MMSE, such as the d2 sustained attention test, are strongly recommended in a future study [56]. Second, although all covariates were treated as time invariant, the possibility that changes in health behaviors and conditions influenced changes in cognitive function cannot be ruled out. This should be further investigated in a future study. Third, we cannot rule out the possibility that the associations observed in this study might be attributed to the multiple cohort effects of the KLoSA. Despite the limitations, our study also had a few strengths. First, this is one of the longest follow-up studies conducted in South Korea involving a representative sample of older Korean adults. Second, this study used a number of potential confounders, including demographics, SES, health behaviors, health conditions, and physical activity and fitness, as predictors of age-related cognitive decline.

## 5. Conclusions

In summary, we found that age-related cognitive decline was multifactorial in older Korean adults, including lower educational attainment, lower income, living in a rural area, UWL, depression, and lower handgrip strength in conjunction with predisposition to cognitive impairment. From a public health perspective, the current findings of the study suggest that multifaceted interventions, targeting lower socio-economic status, health conditions, and unhealthy behaviors, should be implemented as preventive measures against age-related cognitive declines in older Korean adults.

## Figures and Tables

**Table 1:** Descriptive statistics of measured variables among cognitively normal, mild, and moderate/severe cognitive impairments.

Variables		Total (*n* = 727)	Normal Cognition (*n* = 401)	Mild Cognitive Impairment (*n* = 143)	Moderate/Severe Cognitive Impairment (*n* = 183)	*p* Value
Demographics and socioeconomic status						
Age (year)		69.3 ± 3.7	68.7 ± 3.4	69.5 ± 3.7	70.4 ± 4.0	<0.001
Body mass index (kg/m^2^)		23.5 ± 3.2	23.6 ± 2.7	23.0 ± 3.6	23.6 ± 3.7	0.127
Gender, *n* (%)	330 (45.4)	163 (40.6)	62 (43.4)	105 (57.4)	<0.001	<0.001
	397 (54.6)	238 (59.4)	81 (56.6)	78 (42.6)	78 (42.6)	
Existence of spouse, *n* (%)	574 (79.0)	331 (82.5)	110 (76.9)	133 (72.7)	0.006	0.006
	153 (21.0)	70 (17.5)	33 (23.1)	50 (27.3)	50 (27.3)	
Having a religion, *n* (%)	435 (59.8)	235 (58.6)	90 (62.9)	110 (60.1)	0.732	0.732
	292 (40.2)	166 (48.4)	53 (37.1)	73 (39.3)	73 (39.3)	
Residence area, *n* (%)	501 (68.9)	295 (73.6)	86 (60.1)	120 (65.6)	0.048	0.048
	226 (31.1)	106 (26.4)	57 (39.9)	63 (34.4)	63 (34.4)	
Education, *n* (%)	420 (57.8)	197 (49.1)	86 (60.1)	137 (74.9)	<0.001	<0.001
	249 (34.3)	161 (40.1)	49 (34.3)	39 (21.3)	39 (21.3)	
	58 (8.0)	43 (10.7)	8 (5.6)	7 (3.8)	7 (3.8)	
Income (10,000 won)		107 ± 196	123 ± 245	100 ± 120	78 ± 96	0.030
Health behaviors						
Current/past smoking, *n* (%)		232 (31.9)	133 (33.2)	48 (33.6)	51 (27.9)	0.201
Heavy drinking, *n* (%)		79 (10.9)	48 (12.0)	19 (13.3)	12 (6.6)	0.046
Physical inactivity, *n* (%)		423 (58.2)	220 (54.9)	80 (55.9)	123 (67.2)	0.005
Handgrip strength (kg)		26.7 ± 7.5	27.6 ± 7.5	26.9 ± 7.2	24.5 ± 7.2	<0.001
Functional limitations						
K-ADL score		7.04 ± 0.57	7.01 ± 0.21	7.01 ± 0.08	7.13 ± 1.10	0.065
IADL score		10.23 ± 1.08	10.17 ± 0.61	10.15 ± 0.62	10.43 ± 1.87	0.015
Health conditions						
CES-D score		4.88 ± 4.31	4.25 ± 3.94	5.32 ± 4.19	5.91 ± 4.93	<0.001
Experience of falls, *n* (%)		32 (4.4)	17 (4.2)	4 (2.8)	11 (6.0)	0.353
Number of physician-diagnosed comorbidities		0.85 ± 0.90	0.76 ± 0.84	0.89 ± 0.94	1.02 ± 0.98	0.004
Number of medications		0.68 ± 0.81	0.60 ± 0.77	0.75 ± 0.86	0.80 ± 0.85	0.011
Unintentional weight loss, *n* (%)		69 (9.5)	25 (6.2)	17 (11.9)	27 (14.8)	0.001

CES-D: Center for Epidemiologic Studies Depression Scale; K-ADL: Korean Activities of Daily Living; IADL: Instrumental Activities of Daily Living.

**Table 2:** Bivariate correlations between changes in cognitive function and covariates in study participants.

	Cov1	Cov2	Cov3	Cov4	Cov5	Cov6	Cov7	Cov8	Cov9	Cov10	Cov11	Cov12	Cov13	Cvo14	Cov15	Cov16	Cov17	Cov18
R (*p* value)	−0.193 (<0.001)	−0.136 (<0.001)	0.006 (0.586)	−0.085 (<0.001)	−0.013 (0.212)	−0.107 (<0.001)	0.234 (<0.001)	0.152 (<0.001)	−0.071 (<0.001)	0.063 (<0.001)	0.069 (<0.001)	0.185 (<0.001)	−0.017 (0.114)	−0.0114 (<0.001)	−0.029 (0.006)	−0.091 (<0.001)	−0.046 (0.001)	0.033 (0.002)
R^2^	0.037	0.018	0.001	0.007	0.001	0.011	0.054	0.023	0.005	0.004	0.004	0.034	0.001	0.013	0.001	0.008	0.002	0.001
VIFs	1.173	2.783	1.088	1.162	1.118	1.108	1.295	1.061	1.644	1.126	1.143	2.895	1.738	1.100	1.031	2.218	2.073	1.042

Cov1: age; Cov2: sex; Cov3: body mass index; Cov4: absence of spouse; Cov5: having a religion; Cov6: residence area; Cov7: education; Cov8: income; Cov9: smoking; Cov10: heavy drinking; Cov11: physical activity; Cov12: handgrip strength; Cvo13: Korean Activities of Daily Living score; Cov14: Center for Epidemiologic Studies Depression scale; Cov15: experience of falls; Cov16: number of physician-diagnosed comorbidities; Cvo17: number of medications; Cov18: unintentional weight loss. VIFs: variance of inflation factors.

**Table 3:** Parameter estimates of linear mixed regression model for MMSE-based cognitive scores.

			Unadjusted Model			Adjusted Model ^^^		
Parameter		Categories	Estimation	95% CI	*p*	Estimation	95% CI	*p*
Intercept						28.044	24.156~31.933	<0.001
Category of cognitive function (ref: normal cognition)		Moderate/severe cognitive impairment	−4.524	−4.784~−4.264	<0.001	0.686	0.303~1.069	<0.001
		Mild cognitive impairment	−2.405	−2.675~−2.135	<0.001	−0.081	−0.477~0.317	0.691
Time (per year)			−0.494	−0.545~−0.444	<0.001	0.084	0.009~0.160	0.028
Age (per year)			−0.045	−0.074~−0.016	0.003	−0.026	−0.067~0.015	0.220
Body mass index (per kg/m^2^)			0.008	−0.025~0.041	0.636	0.010	−0.037~0.057	0.689
Presence of spouse (ref: no)		Yes	0.090	−0.187~0.367	0.523	−0.096	−0.448~0.297	0.632
Religion (ref: no)		Yes	0.130	−0.091~0.351	0.250	0.185	−0.129~0.498	0.247
Residence (ref. village)		Town or better	0.398	0.157~0.638	0.001	0.298	−0.043~0.638	0.087
Education (ref. college)		Elementary or less	−1.159	−1.576~−0.742	<0.001	−1.126	−1.717~−0.535	<0.001
		Middle/high school	−0.642	−1.042~−0.242	0.026	−0.605	−1.172~−0.037	0.037
Income (per won)			0.001	0.001~0.001	0.017	0.001	0.000~0.002	0.016
Current/past smokers (ref. yes)		No	−0.238	−0.508~0.032	0.084	−0.010	−0.393~0.372	0.957
Heavy drinking (ref. yes)		No	−0.431	−0.776~−0.086	0.014	−0.389	−0.878~0.100	0.118
UWL (ref. yes)		No	−0.592	−0.942~−0.242	0.001	−0.735	−1.231~−0.239	0.004
Fall experience (ref. yes)		No	−0.682	−1.186~−0.178	0.008	−0.458	−1.172~0.256	0.208
Number of diagnosed diseases (ref: 0)			0.065	−0.170~0.301	0.586	0.017	−0.317~0.350	0.992
Number of medications (ref: 0)			−0.143	−0.414~0.119	0.285	−0.163	−0.534~0.208	0.388
Physical activity (ref. inactive)		Active	0.080	−0.146~0.306	0.489	0.202	−0.118~0.253	0.216
Handgrip strength (per kg)			0.034	0.011~0.056	0.004	0.039	0.007~0.072	0.018
CES_D score			−0.008	−0.033~0.017	0.552	−0.018	−0.054 ~0.017	0.319
KADL score			−0.183	−0.359~−0.007	0.042	−0.026	−0.274~−0.274	0.211
Category of cognitive function × time (ref. normal cognition)		Moderate/severe cognitive impairment × time				−1.740	−1.877~−1.604	<0.001
		Mild cognitive impairment × time				−0.775	−0.922~−0.628	<0.001
Random variance								
	Intercept				0.472	0.184~1.209	0.037	0.037
	Linear slope				0.380	0.195~0.566	<0.001	<0.001
	Residual				7.675	7.354~8.011	<0.001	<0.001

^^^ Adjusted model included random effects (intercept and slope) and interaction between cognitive function and time.

## Data Availability

The data presented in this study are available on request from the corresponding author. The data are not publicly available to protect confidentiality of the research participants.
